# Two novel species of *Neohelicomyces* (Tubeufiaceae, Tubeufiales) from Guizhou Province, China

**DOI:** 10.3897/mycokeys.129.178173

**Published:** 2026-03-02

**Authors:** Gui-Li Zhao, Xing-Juan Xiao, Shan-Shan Song, Samantha C. Karunarathna, Xue-Yan Chen, Ying Liu, Yong-Zhong Lu, Ning-Guo Liu

**Affiliations:** 1 School of Chemical Engineering, Guizhou Institute of Technology, Guiyang 550025, China Guizhou Key Laboratory of Agricultural Microbiology, Guizhou Academy of Agricultural Sciences Guiyang China; 2 Guizhou Key Laboratory of Agricultural Microbiology, Guizhou Academy of Agricultural Sciences, Guiyang 550009, China Center for Yunnan Plateau Biological Resources Protection and Utilization, College of Biological Resource and Food Engineering, Qujing Normal University Qujing China; 3 School of Food and Pharmaceutical Engineering, Guizhou Institute of Technology, Guiyang 550025, China School of Chemical Engineering, Guizhou Institute of Technology Guiyang China; 4 Department of Health Management, Zunyi Medical and Pharmaceutical College, Zunyi 563006, China School of Food and Pharmaceutical Engineering, Guizhou Institute of Technology Guiyang China; 5 Center for Yunnan Plateau Biological Resources Protection and Utilization & Yunnan International Joint Laboratory of Fungal Sustainable Utilization in South and Southeast Asia, College of Biology and Food Engineering, Qujing Normal University, Qujing 655099, China Department of Health Management, Zunyi Medical and Pharmaceutical College Zunyi China

**Keywords:** 2 new taxa, Dothideomycetes, helicosporous hyphomycetes, phylogeny, taxonomy

## Abstract

During a survey of microfungal diversity in Guizhou Province, southwestern China, four helicosporous taxa were collected and isolated from decaying wood in both freshwater and terrestrial habitats. Phylogenetic analyses based on LSU, ITS, *tef*1-α, and *rpb*2 sequences, along with morphological assessments, revealed two new species: *Neohelicomyces saprobicus* and *N. uniramulosus*. Morphologically, *Neohelicomyces saprobicus* differs from its close relative *N. sexualis* by its asexual mode of reproduction, in contrast to the sexual reproduction observed in *N. sexualis*. *Neohelicomyces uniramulosus* is distinguished from its close relative *N. helicosporus* and *N. hyalosporus* by its conidiophores. Detailed morphological descriptions, illustrations, and taxonomic notes are provided. In addition, the present study summarizes the main morphological characteristics, host associations, and geographical distributions of *Neohelicomyces*.

## Introduction

Guizhou Province, situated in southwestern China, harbors an exceptionally rich diversity of vegetation, encompassing ecosystems that range from subtropical evergreen broad-leaved forests in karst landscapes to alpine coniferous forests at high elevations (Hong et al. 2025). The region’s pronounced altitudinal gradients and complex topography have given rise to a wide spectrum of microhabitats, creating ideal ecological conditions for fungal colonization and diversification (Zhu et al. 2022; Zou et al. 2022). Consequently, Guizhou has witnessed particularly active research and field investigations on helicosporous hyphomycetes in recent years (Lu et al. 2018; Liu et al. 2024; Ma et al. 2024a).

*Neohelicomyces*, a genus of helicosporous hyphomycetes affiliated with Tubeufiaceae, was established by Luo et al. (2017), with *N. aquaticus* designated as the type species. Currently, there are 38 species of the genus in the Index Fungorum (https://www.indexfungorum.org/), each supported by molecular data (Table 1). Species within *Neohelicomyces* have a wide distribution, with records from Asia, Europe, and North America (Luo et al. 2017; Lu et al. 2018, 2022; Tibpromma et al. 2018; Crous et al. 2019a, 2019b; Dong et al. 2020; Hsieh et al. 2021; Yang et al. 2023; Ma et al. 2025; Peng et al. 2025). The asexual morph of *Neohelicomyces* is primarily characterized by its coiled conidia, along with macronematous, mononematous, branched and/or unbranched conidiophores, monoblastic to polyblastic, integrated, terminal or intercalary conidiogenous cells (Luo et al. 2017; Lu et al. 2018, 2022; Tibpromma et al. 2018; Crous et al. 2019a, 2019b; Ma et al. 2024a, 2024b; Dong et al. 2020; Hsieh et al. 2021; Yang et al. 2023; Ma et al. 2025). To date, only one species with a sexual morph has been discovered within *Neohelicomyces*, characterized by superficial, solitary, scattered, subglobose, brown, ascomata without setae; asci 8-spored, bitunicate, cylindric-clavate, rounded at apex, short pedicelate; ascospores multi-seriate, narrowly cylindrical, straight to slightly curved, hyaline to pale brown, septate, not constricted at septa, rough (Sun et al. 2025).

**Table 1.** Taxa used in the phylogenetic analysis of this study, along with their corresponding GenBank accession numbers.

Taxon	Strain	LSU	ITS	*tef*1-α	*rpb*2
*Bezerromyces brasiliensis*	CBS 141545	NG_069376	NR_153463	–	–
*Bezerromyces brasiliensis*	URM7411	KX518623	KX470390	KX518631	–
*Muripulchra aquatica*	DLUCC 0571	KY320548	KY320531	–	–
*Muripulchra aquatica*	MFLUCC 15-0249^T^	KY320549	KY320532	–	–
*Neohelicomyces acropleurogenus*	CGMCC 3.25549^T^	PP639450	PP626594	PP596351	PP596478
*Neohelicomyces aquaticus*	MFLUCC 16-0993^T^	KY320545	KY320528	KY320561	MH551066
*Neohelicomyces aquisubtropicus*	GZCC 23-0080^T^	PQ098537	PQ098499	PV768327	PV768336
*Neohelicomyces aseptatus*	CGMCC 3.25564^T^	PP639451	PP626595	PP596352	PP596479
*Neohelicomyces astrictus*	HKAS 105122^T^	PQ898796	PQ898760	PV040811	–
*Neohelicomyces brunneus*	HKAS 105147^T^	PQ898805	PQ898768	PV040818	–
*Neohelicomyces coffeae*	GMBCC 2225^T^	PX308848	PX308843	PX314510	PX314514
*Neohelicomyces dehongensis*	MFLUCC 18-1029^T^	MN913709	NR_171880	MT954393	–
*Neohelicomyces denticulatus*	GZCC 19-0444^T^	MW133855	OP377832	–	–
*Neohelicomyces deschampsiae*	CPC 33686^T^	MK442538	MK442602	–	–
*Neohelicomyces edgeworthiae*	CGMCC 3.25565^T^	PP639453	PP626597	PP596354	PP596481
*Neohelicomyces grandisporus*	KUMCC 15-0470^T^	KX454174	KX454173	–	MH551067
*Neohelicomyces guizhouensis*	GZCC 23-0725^T^	PP512973	PP512969	PP526727	PP526733
*Neohelicomyces guttulatus*	CGMCC 3.25550^T^	PP639454	PP626598	PP596355	–
*Neohelicomyces hainanensis*	GZCC 22-2009^T^	OP508774	OP508734	OP698085	OP698074
*Neohelicomyces helicosporus*	GZCC 23-0633^T^	PP512975	PP512971	PP526729	PP526735
*Neohelicomyces hyalosporus*	GZCC 16-0086^T^	MH558870	MH558745	MH550936	MH551064
*Neohelicomyces hydei*	GZCC 23-0727^T^	PP512977	–	PP526731	PP526737
*Neohelicomyces lignicola*	CGMCC 3.25551^T^	PP639456	PP626600	PP596357	PP596483
*Neohelicomyces longisetosus*	NCYU 106H1.1.1^T^	–	MT939303	–	–
*Neohelicomyces macrosporus*	CGMCC 3.25552^T^	PP639457	PP626601	PP596358	PP596484
*Neohelicomyces maolanensis*	GZCC 23-0079^T^	PQ098529	–	PQ490683	PQ490677
*Neohelicomyces melaleucae*	CPC 38042^T^	MN567661	MN562154	MN556835	–
*Neohelicomyces pallidus*	CBS 271.52	AY856887	AY916461	–	–
*Neohelicomyces pallidus*	CBS 962.69	AY856886	AY916460	–	–
*Neohelicomyces pandanicola*	KUMCC 16-0143^T^	MH260307	MH275073	MH412779	–
*Neohelicomyces puerensis*	GMBCC 2217^T^	PX308846	PQ737369	PX314508	PX314512
*Neohelicomyces qixingyaensis*	CGMCC 3.25569^T^	PP639458	PP626602	PP596359	PP596485
***Neohelicomyces saprobicus***	**GZCC 23-0743^T^**	**PX625152**	**PX625156**	**PX830980**	–
***Neohelicomyces saprobicus***	**GZCC 23-0744**	**PX625153**	**PX625157**	**PX830981**	–
*Neohelicomyces sexualis*	GUCC 24-0110^T^	PQ570861	PQ570844	PQ761136	–
*Neohelicomyces submersus*	MFLUCC 16-1106^T^	KY320547	KY320530	–	MH551068
*Neohelicomyces subtropicus*	GZCC 23-0076^T^	PQ098530	PQ098492	PQ490685	PQ490679
*Neohelicomyces terrestris*	GZCC 23-0399^T^	PQ098531	PQ098493	–	–
*Neohelicomyces thailandicus*	MFLUCC 11-0005^T^	MN913696	NR_171882	–	–
*Neohelicomyces tropicus*	GZCC 25-0661^T^	PX575664	PX575641	PX512847	PX512838
***Neohelicomyces uniramulosus***	**GZCC 25-0750^T^**	**PX625154**	**PX625158**	–	–
***Neohelicomyces uniramulosus***	**GZCC 25-0751**	**PX625155**	**PX625159**	–	–
*Neohelicomyces wuzhishanensis*	GZCC 23-0410^T^	PQ098532	PQ098494	PV768325	PV768334
*Neohelicomyces xiayadongensis*	CGMCC 3.25568^T^	PP639460	PP626604	PP596361	PP596487
*Neohelicomyces yunnanensis*	GZCC 23-0735^T^	PP664113	PP664109	–	–
*Neohelicosporium bambusicola*	MFLUCC 21-0156^T^	OL606146	OL606157	OL964517	OL964523
*Neohelicosporium ellipsoideum*	MFLUCC 16-0229^T^	MH558873	MH558748	MH550939	MH551071
*Neohelicosporium fusisporum*	MFUCC 16-0642^T^	MG017613	MG017612	MG017614	–
*Neohelicosporium guineensis*	ZHKUCC 24-0113^T^	PP860102	PP860090	PP858062	PP858074
*Neohelicosporium hyalosporum*	GZCC 16-0076^T^	MF467936	MF467923	MF535249	MF535279
*Neohelicosporium irregulare*	MFLUCC 17-1796^T^	MH558877	MH558752	MH550943	MH551075
*Neohelicosporium krabiense*	MFLUCC 16-0224^T^	MH558879	MH558754	MH550945	MH551077
*Neohelicosporium laxisporum*	MFLUCC 17-2027^T^	MH558880	MH558755	MH550946	MH551078
*Tubeufia guttulata*	GZCC 23-0404^T^	OR030834	OR030841	OR046678	OR046684
*Tubeufia guttulata*	GZCC 23-0590	OR066420	OR066413	OR058859	OR058852
*Tubeufia hainanensis*	GZCC 22-2015^T^	OR030835	OR030842	OR046679	OR046685
*Tubeufia javanica*	MFLUCC 12-0545^T^	KJ880036	KJ880034	KJ880037	–
*Tubeufia krabiensis*	MFLUCC 16-0228^T^	MH558917	MH558792	MH550985	MH551118
*Tubeufia latispora*	MFLUCC 16-0027^T^	KY092412	KY092417	KY117033	MH551119
*Tubeufia laxispora*	MFLUCC 16-0232^T^	KY092408	KY092413	KY117029	MF535287
*Tubeufia machaerinae*	MFLUCC 17-0055	MH558920	MH558795	MH550988	MH551122
*Tubeufia mackenziei*	MFLUCC 16-0222^T^	KY092410	KY092415	KY117031	MF535288
*Tubeufia muriformis*	GZCC 22-2039^T^	OR030836	OR030843	OR046680	OR046686
*Tubeufia nigroseptum*	CGMCC 3.20430^T^	MZ853187	MZ092716	OM022002	OM022001
*Tubeufia pandanicola*	MFLUCC 16-0321^T^	MH260325	MH275091	–	–

Note: The newly generated sequences are in bold black. “^T^” indicates the type strains. “–” indicates sequence unavailable.

While conducting a survey focused on documenting fungal diversity in the freshwater and terrestrial habitats of Guizhou Province, China, we collected four helicosporous fungal taxa. We aim to 1) characterize the morphological traits and provide illustrations of these collections, 2) assess their phylogenetic positions using multi-gene phylogenetic analyses, and 3) formally describe novel species identified based on morphological and phylogenetic evidence. Accordingly, *Neohelicomyces saprobicus* sp. nov. and *N. uniramulosus* sp. nov. are described and introduced herein.

## Materials and methods

### Specimen collection, examination, and isolation

Moist decaying wood samples were collected in Guizhou Province, China, and immediately sealed in sterile airtight bags. Once the samples were taken back to the laboratory, single conidium isolations were conducted following the protocol outlined by Senanayake et al. (2020). Germinating conidia were aseptically transferred to fresh potato dextrose agar (PDA) medium and incubated at room temperature to promote colony development. Colony morphology, including color, shape, and texture, was recorded. Fungal colonies and micromorphological structures on natural substrate were observed with a Nikon SMZ 745 and SMZ 800N dissecting microscopes (Nikon, Tokyo, Japan). The microscopic morphology of fruiting bodies was documented with a Nikon ECLIPSE microscope equipped with a DS-Ri2 camera. Pure cultures were deposited in the Guizhou Culture Collection (GZCC), Guiyang, China, and dried vouchers were stored in the Herbarium of Cryptogams, Kunming Institute of Botany, Academia Sinica (HAKS), Kunming, China, and Herbarium of Guizhou Academy of Agriculture Sciences (GZAAS), Guiyang, China. The novel species were registered in the Fungal Names (https://nmdc.cn/fungalnames/registe) database.

### DNA extraction, PCR amplification, and sequencing

Fresh fungal mycelia were carefully scraped using a sterilized toothpick and transferred into a 1.5 mL microcentrifuge tube. Genomic DNA was extracted using the Ezup Column Fungi Genomic DNA Purification Kit (Sangon Biotech, Shanghai, China), following the manufacturer’s instructions. Three primer pairs were employed to amplify specific gene regions: LR0R/LR5 (Vilgalys and Hester 1990) for the large subunit ribosomal RNA (LSU); ITS5/ITS4 (White et al. 1990) for the internal transcribed spacer (ITS); EF1-983F/EF1-2218R (Rehner and Buckley 2005) for translation elongation factor 1-alpha (*tef*1-α). Polymerase chain reaction (PCR) was performed in a final volume of 50 μL, comprising 2 μL of DNA, 2 μL each of forward and reverse primers, and 44 μL of 1.1× T3 Super PCR Mix (Qingke Biotech, Chongqing, China). The PCR conditions followed the protocols described by Ma et al. (2024a) and Zhao et al. (2025, 2026). Amplified products were purified and sequenced by Tsingke Biotechnology Co., Ltd. (Beijing, China).

### Phylogenetic analyses

The sequence data of the newly obtained taxa were checked using BioEdit Sequence Alignment software. Forward and reverse sequence data were assembled with SeqMan v.7.0.0 (DNASTAR, Madison, WI, USA) (Clewley 1995). Sequences listed in Table 1 were downloaded from GenBank (https://www.ncbi.nlm.nih.gov/) based on BLASTn search results and recently published articles. The single-gene datasets were aligned using the online MAFFT version 7 (https://mafft.cbrc.jp/alignment/server/) (Katoh and Standley 2013), followed by trimming with trimAl v.1.2 (Capella-Gutiérrez et al. 2009). The concatenation of single-gene alignments was performed using Sequence Matrix v.1.7.8 (Vaidya et al. 2011). Maximum Likelihood (ML) analyses were conducted on the CIPRES Science Gateway platform (Miller et al. 2012) (https://www.phylo.org/portal2/home.action) using RAxML-HPC2 8.2.12 on XSEDE with the GTRGAMMA model. Bayesian Inference (BI) was carried out using MrBayes v.3.2.7a on XSEDE, which employs the Markov chain Monte Carlo (MCMC) algorithm (Ronquist et al. 2012). Maximum likelihood bootstrap support (ML-BS) and Bayesian posterior probabilities (BYPP) are shown above each branch. Phylogenetic trees were visualized using FigTree v.1.4.4, and the final layout of figures was edited in Adobe Illustrator CC 2019 (version 23.1.0; Adobe Systems, USA).

## Results

### Phylogenetic analysis results

**Figure 1.**
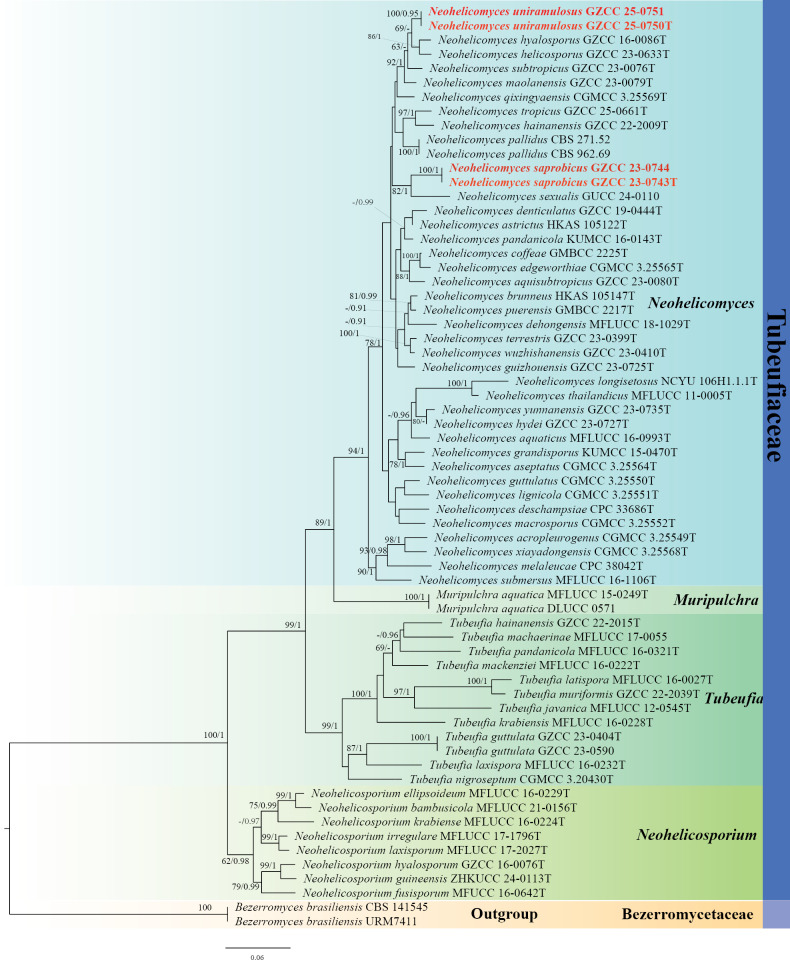
The ML tree based on a combined dataset of LSU, ITS, *tef*1-α, and *rpb*2 sequences. Bootstrap support values for ML greater than 60% and BYPP greater than 0.90 are indicated near each node as ML-BS and BYPP, respectively. ^“^T^”^ denotes ex-type isolates. Newly generated isolates are highlighted in bold red.

The concatenated dataset comprised 65 strains, including newly collected taxa. ML analysis of the combined dataset yielded the best-scoring tree (Fig. 1), with a final ML optimization likelihood score of -22330.459568. The alignment spanned a total of 3,326 characters (including gaps), partitioned as follows: LSU (1–844), ITS (845–1,379), *tef*1-α (1,380–2,281), *rpb*2 (2,282–3,326). The tree was rooted with *Bezerromyces brasiliensis* (CBS 141545 and URM7411). The alignment matrix revealed 1,154 unique site patterns, with gaps and undetermined characters accounting for 23.37% of the dataset. The topologies derived from ML and BI analyses were congruent in overall structure.

In the phylogenetic tree (Fig. 1), our two strains of *Neohelicomyces saprobicus* (GZCC 23-0743 and GZCC 23-0744) formed a well-supported, distinct clade (100% ML-BS, 1.00 BYPP), which is closely related to *N. sexualis* (82% ML-BS, 1.00 BYPP). Similarly, our two strains of *N. uniramulosus* (GZCC 25-0750 and GZCC 25-0751) formed a distinct clade with 100% ML-BS and 0.95 BYPP support, positioned as a sister group to the clade comprising *N. helicosporus* (GZCC 23-0633) and *N. hyalosporus* (GZCC 16-0086).

### Taxonomy

#### Neohelicomyces saprobicus

Taxon classificationFungiTubeufialesTubeufiaceae

G.L. Zhao, X.J. Xiao, N.G. Liu & Y.Z. Lu sp. nov.

2FA5B65E-53F7-59DD-AB71-88AB89F9218B

Fungal Names: FN 573086

Fig. 2

##### Etymology.

The epithet “*saprobicus*” is derived from saprobic, referring to the saprobic lifestyle of the fungus.

##### Holotype.

GZAAS 23-0826.

##### Description.

***Saprobic*** on submerged decaying wood in a freshwater lake. ***Sexual morph*** Undetermined. ***Asexual morph*** Hyphomycetous, helicosporous. ***Colonies*** on natural substrate superficial, effuse, gregarious, white, consisting of a dense, glistening aggregation of conidia. ***Mycelium*** partly immersed, partly superficial, hyaline to pale brown, smooth. ***Conidiophores*** 126–160 μm long, 4.5–6 μm wide (x̄ = 144 × 5.5 μm, n = 25), macronematous, mononematous, erect, flexuous, cylindrical, branched, hyaline to pale brown, smooth, thick-walled. ***Conidiogenous cells*** 7–18 × 3.0–5.0 µm (x̄ = 13.5 × 3.5 µm, n = 25), holoblastic, mono- to polyblastic, integrated, sympodial, terminal or intercalary, cylindrical, with denticles (2.0–4.0 μm long, 2.0–2.5 μm wide), hyaline to brown, smooth-walled. ***Conidia*** 140–195 µm long × 2.5–3.5 µm wide (x̄ = 170 × 3.0 µm, n = 30) solitary, acropleurogenous, helicoid, 25–39 μm in diameter (x̄ = 33 μm, n = 15), with a taper toward the rounded ends, growing on dentate protrusion, obscure multiseptate, loosely coiled 1³/4–3 times, turning into loose coils in water, guttulate, hyaline, smooth-walled.

**Figure 2.**
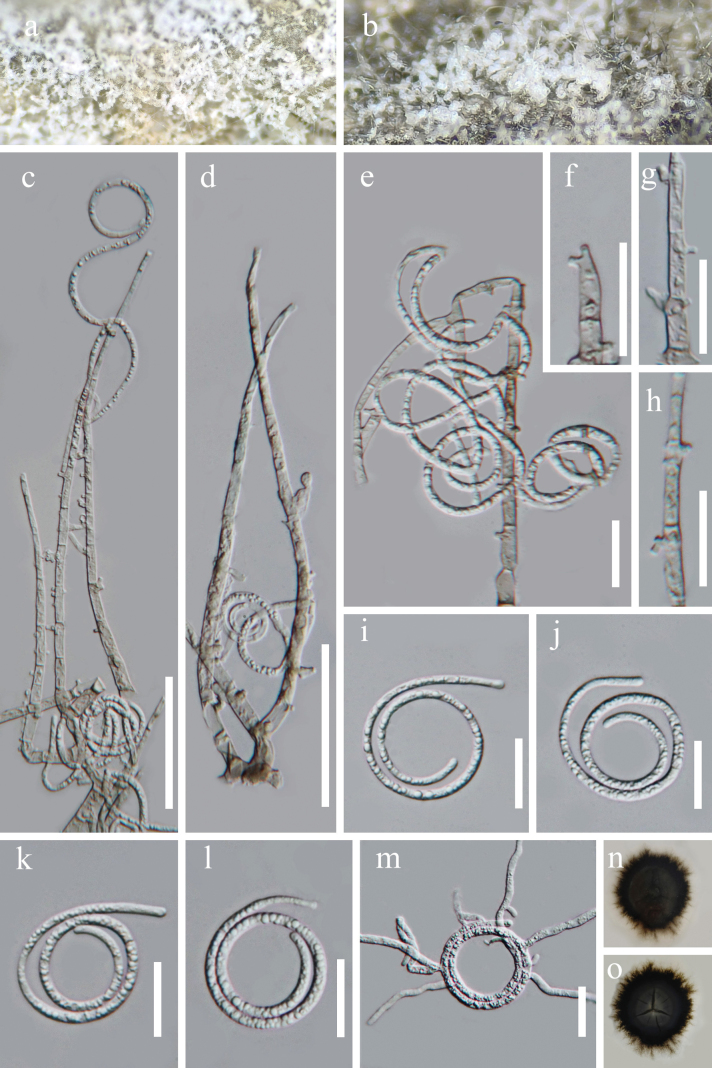
*Neohelicomyces saprobicus* (GZAAS 23-0826, holotype). **a, b**. Colonies on the host surface; **c–e**. Conidiophores and conidia; **f–h**. Conidiogenous cells; **i–l**. Conidia; **m**. Germinated conidium; **n, o**. Surface and reverse colonies on PDA after 3 months of incubation at 25 °C. Scale bars: 50 μm (**c–d**); 20 μm (**e–m**).

##### Culture characteristics.

Conidia germinated on PDA and produced germ tubes within 24 h at 25 °C. The colony reached a diameter of 3.7 cm, after 3 months of incubation at room temperature, circular, convex at the center, and undulate at the margin, dense in texture, brown to dark brown.

##### Material examined.

China • Guizhou Province, Anshun City, Puding County, Yelang Lake Reservoir (26°21'48"N, 105°45'55"E, 1111 m), on submerged decaying wood in a freshwater lake, 21 June 2025, Xing-Juan Xiao, YLS24a (GZAAS 23-0826, holotype), ex-type culture GZCC 23-0743; *ibid*., YLS24b (GZAAS 23-0827, isotype), ex-isotype culture GZCC 23-0744.

##### Notes.

In the phylogenetic analyses (Fig. 1), the strains of *Neohelicomyces saprobicus* (GZCC 23-0743 and GZCC 23-0744) formed a distinct clade, sister to *N. sexualis* with 82% ML-BS and 1.00 BYPP support. Comparison of nucleotide sequences between *N. saprobicus* (GZCC 23-0743) and *N. sexualis* (GUCC 24-0110) revealed nucleotide base differences of 10/701 bp (1%, including 0 gap) in the LSU, 26/360 bp (6%, including 5 gaps) in the ITS, and 25/877 bp (3%, including 0 gap) in the *tef*1-α. As direct morphological comparison between *N. saprobicus* (asexual morph) and *N. sexualis* (sexual morph) is unwarranted (Sun et al. 2025), we introduce *N. saprobicus* as a new species based on phylogenetic analyses.

#### Neohelicomyces uniramulosus

Taxon classificationFungiTubeufialesTubeufiaceae

G.L. Zhao, X.J. Xiao, N.G. Liu & Y.Z. Lu sp. nov.

A0C1D4F8-AEA6-5F02-85AE-918C01DEE931

Fungal Names: FN 573087

Fig. 3

##### Etymology.

Refers to the basally branched conidiophores of the fungus.

##### Holotype.

HKAS 149897.

##### Description.

***Saprobic*** on decaying wood in a terrestrial habitat. ***Sexual morph*** Undetermined. ***Asexual morph*** Hyphomycetous, helicosporous. ***Colonies*** on natural substrate superficial, effuse, gregarious, white, composed of a dense, glistening aggregation of conidia. ***Mycelium*** consists of partly immersed, partly superficial, composed of septate, branched hyphae, hyaline to pale brown, smooth. ***Conidiophores*** (33–)75–205(–220) μm long, (2.5–)3.5–5.5(–7.0) μm wide (x̄ = 140 × 4.5 μm, n = 40), macronematous, mononematous, erect, flexuous, cylindrical, basally branched, up to 16 septate, pale brown to brown, smooth, thick-walled. ***Conidiogenous cells*** 8–18 × 3–5 µm (x̄ = 13.5 × 3.8 µm, n = 35), holoblastic, monoblastic to polyblastic, integrated, sympodial, intercalary or terminal, cylindrical, denticulate, truncate at apex after conidial secession, hyaline to brown, smooth-walled. ***Conidia*** (115–)125–152(–166) µm long × 2–3.5 µm wide (x̄ = 139 × 2.7 µm, n = 25), solitary, acropleurogenous, helicoid, rounded at the tip, 13–23 μm in diameter (x̄ = 18 μm, n = 15), with indistinctly multiseptate, tightly coiled 3¹/3–34/5 times, becoming loosely coiled when in water, guttulate, hyaline, smooth-walled.

**Figure 3.**
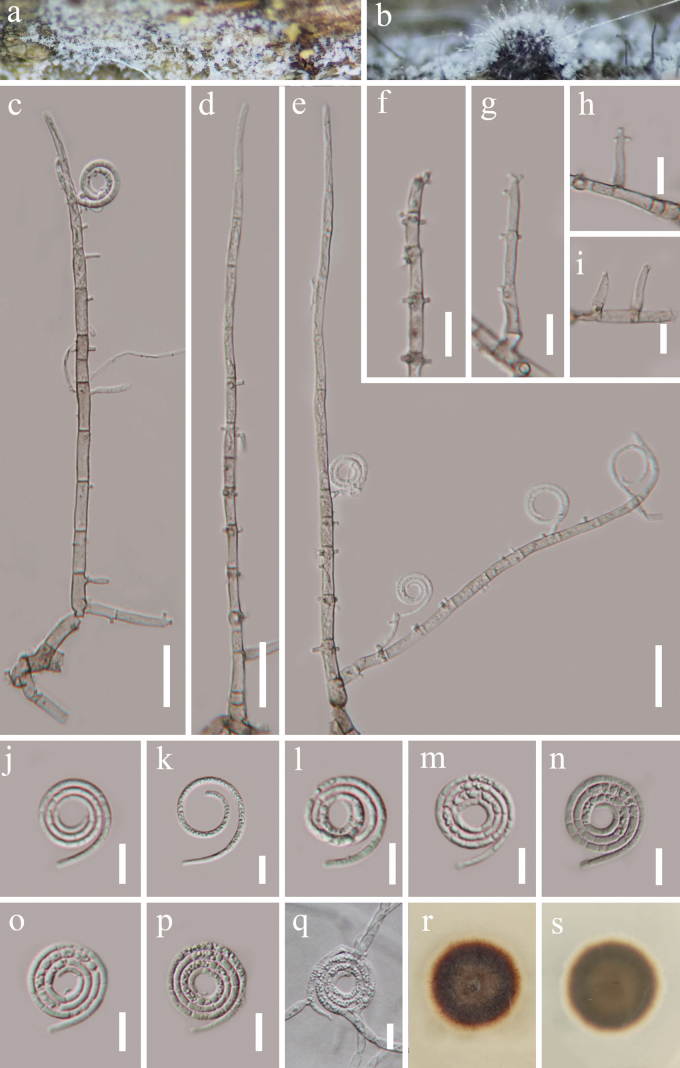
*Neohelicomyces uniramulosus* (HKAS 149897, holotype). **a, b**. Colonies on the host surface; **c–e**. Conidiophores, conidiogenous cells and conidia; **f–i**. Conidiogenous cells; **j–p**. Conidia; **q**. Germinated conidium; **r, s**. Surface and reverse colonies on PDA after 32 days of incubation at 25 °C. Scale bars: 20 μm (**c–e**); 10 μm (**f–q**).

##### Culture characteristics.

Germination of conidia was noted within 24 hours under incubation on PDA medium at 28 °C. After 1 month of incubation at room temperature, the colony reached a diameter of 2.2 cm, circular, smooth margin, dense in texture, top view of colony brown to dark brown, reverse light brown to dark brown.

##### Material examined.

China • Guizhou Province, Qiandongnan Miao and Dong Autonomous Prefecture, Zhenyuan County, Gaoguohe Scenic Area (27°14'40"N, 108°18'30"E, 631 m), on decaying wood in a terrestrial habitat, 3 May 2025, Xing-Juan Xiao, GF29a (HKAS 149897, holotype), ex-type culture GZCC 25-0750; *ibid*., GF29b (paratype, HKAS 149898), ex-paratype culture GZCC 25-0751.

##### Notes.

In our phylogenetic analyses, *Neohelicomyces uniramulosus* (GZCC 25-0750 and GZCC 25-0751) formed an independent clade and is sister to *N. helicosporus* (GZCC 23-0633) and *N. hyalosporus* (GZCC 16-0086) (Fig. 1). Comparison of ITS and LSU sequence between *N. uniramulosus* (GZCC 25-0750) and *N. helicosporus* (GZCC 23-0633), revealed nucleotide base differences of 34/516 bp (6.6%, including 10 gaps), 1/829 bp (0.1%, including 0 gap), respectively. Similarly, comparison between *N. uniramulosus* (GZCC 25-0750) and *N. hyalosporus* (GZCC 16-0086) showed nucleotide base differences of 26/506 bp (5%, including ten gaps), 1/825 bp (0.1%, including 0 gap), respectively. Morphologically, *N. uniramulosus* can be distinguished from *N. helicosporus* by the structure of their conidiophores: the former possesses branched conidiophores, whereas the latter has unbranched ones (Ma et al. 2024a). Compared to *N. hyalosporus*, *N. uniramulosus* has shorter conidiophores (75–205 µm vs. 210–290 µm) (Lu et al. 2018). Consequently, combining phylogenetic analyses and morphological assessments, we introduce *N. uniramulosus* as a novel species.

## Discussion

In this study, two novel species, namely *Neohelicomyces saprobicus* and *N. uniramulosus*, are introduced. These findings enhance the species diversity of the genus *Neohelicomyces*.

To date, 40 species (including two new species in this study) within *Neohelicomyces* have been reported, including 36 species from China, one species each in Germany, Thailand, and the United States, and one species with a cosmopolitan distribution (Table 2). The specific distribution within China includes Guizhou Province (16 species), Yunnan Province (eight species), Hainan Province (eight species), Guangxi Zhuang Autonomous Region (two species), Taiwan Province (one species), and Xizang Autonomous Region (one species) (Table 2). China currently harbors the highest known diversity and concentration of *Neohelicomyces* species, with the southwestern (Guizhou and Yunnan) and southern (Hainan) regions representing domestic biodiversity hotspots for this genus (Luo et al. 2017; Ma et al. 2024b; Ma et al. 2025; Gao et al. 2025; Peng et al. 2025; Sun et al. 2025). However, it remains uncertain whether the observed distribution hotspots are primarily driven by natural factors such as climate and habitat heterogeneity, reflect biases resulting from uneven sampling efforts and regional disparities in research intensity, or are influenced by the fact that researchers focusing on this fungal group are mostly concentrated in these regions. Therefore, comprehensive specimen collection and geographically expanded surveys are essential to accurately assess the underlying causes of the current distribution patterns observed in this genus and to draw robust, evidence-based conclusions.

**Table 2.** Morphological characteristics, hosts, and location information of species of *Neohelicomyces*.

Species	Sampling location	Host	Habitat	Conidiophores	Conidia	Conidiogenous cells	Coil times	Reference
*N. acropleurogenus*	China, Hainan Province	decaying wood	terrestrial	20–96 × 3–6 μm	23–25 μm diam. 100–457 × 2–6.5 μm	9.5–16 × 3–6 μm	tightly coiled 3¹/2 times or loosely coiled 1–2 times	Ma et al. (2024b)
*N. aquaticus*	China, Yunnan Province	decaying wood	freshwater	240.5–335.5 × 5–6 μm	154.5–179.5 × 2.5–3.5 μm		2–2¹/2 times, tightly to loosely coiled	Luo et al. (2017)
*N. aquisubtropicus*	China, Guizhou Province	decaying wood	terrestrial	144–193.5 × 3.5–6.5 μm	14.5–17 μm diam. 82.5–126.5 × 2–4 μm	11.5–15 × 3.5–5 μm	tightly coiled up to 3¹/2 times	Ma et al. (2025)
*N. aseptatus*	China, Guizhou Province	decaying wood	terrestrial	117–233.5 × 4–7 μm	13.5–17.5 μm diam. 90–130 × 1.5–2.5 μm	7–16 × 2–5.5 μm	tightly coiled 3¹/5–3¹/4 times	Ma et al. (2024b)
*N. astrictus*	China, Guizhou Province	decaying wood	terrestrial	22–161 × 2.6–5.3 μm	14.5–19.0 μm diam. 100–136.5 × 1.8–3.7 μm	10–17 × 2.5–5.1 μm	tightly coiled 2–3 times	Lin et al. (2025)
*N. brunneus*	China, Guizhou Province	decaying wood	terrestrial	33–217 × 2.5–5.3 μm	14–22 μm diam. 81.5–124.5 × 1.5–2.8 μm	9–17.5 × 2.5–4.4 μm	tightly coiled 2.5–3.5 times	Lin et al. (2025)
*N. coffeae*	China, Yunnan Province	*Coffea arabica*	terrestrial	144.5–274 × 3.4–6.2 μm	13.5–19.4 μm diam. 105.3–146.4 × 2.1–3.3 μm	13.2–20.6 × 3.4–5.2 μm	tightly coiled 2.5–3.75 times	Han et al. (2026)
*N. davidii*	China, Guizhou Province	decaying wood	freshwater	52–169 × 4–7 μm	200–273 × 3–4 μm	9.5–18 × 2–6 μm	loosely coiled 1¹/2–2¹/3 times	Xiao et al. (2025)
*N. dehongensis*	China, Yunnan Province	decaying wood	freshwater	120–250 × 3.3–3.9 µm	20–25 µm diam. 145–210 × 2.2–4 µm	12–17.5 × 3–4 µm	tightly coiled 2.75–3.75 times	Dong et al. (2020)
*N. denticulatus*	China, Guizhou Province	decaying wood	freshwater	111–236 × 3–6.5 μm	16–22 μm diam. 83–121 × 1.5–2.5 μm		tightly coiled 2¹/2–3¹/2 times	Yang et al. (2023)
*N. deschampsiae*	Germany	culm base of dead leaf sheath of *Deschampsia cespitosa* (Poaceae)	terrestrial	150–220 × 3–4 µm	19–22 µm diam. cells 2–2.5 µm diam.	2–5 × 1.5–2.5 µm	coiled 2–3 times	Crous et al. (2019a)
*N. edgeworthiae*	China, Guizhou Province	dead twigs of *Edgeworthia chrysantha*	terrestrial	up to 293.5 × 5.5 μm	21.5–34 μm diam. 121–177 × 2.5–3.5 μm	9–14 × 3.5–4 μm		Ma et al. (2024b)
*N. grandisporus*	China, Yunnan Province	decaying wood	freshwater	107–161 × 4–5 μm	165–214 × 4.5–5.5 μm		loosely coiled 1–1¹/2 times	Luo et al. (2017)
*N. guizhouensis*	China, Guizhou Province	decaying wood	freshwater	78–288 × 4–6 μm	18–21.5 μm diam. 94.5–148.5 × 2–2.7 μm	9–18 × 2.5–4.5 μm	coiled 2³/4–3¹/2 times	Ma et al. (2024a)
*N. guttulatus*	China, Hainan Province	decaying wood	freshwater	61–102 × 3.5–5 μm	24.5–29 μm diam. 158–224 × 2.5–3.5 μm	7.5–17 × 2–4 μm	tightly coiled 2¹/5–3 times	Ma et al. (2024b)
*N. hainanensis*	China, Hainan Province	decaying wood	terrestrial	137–197 × 2.5–5 µm	14–21 µm diam. 1.5–3 µm wide	11–17 × 3–4 µm	coiled 2¹/2–3³/4 times	Lu et al. (2022)
*N. helicosporus*	China, Guizhou Province	decaying wood	terrestrial	105–199 × 3–5.5 μm	15.5–18 μm diam. 103–170 × 2.5–5 μm	13–22 × 2.5–4.5 μm	coiled up to 3³/4 times	Ma et al. (2024a)
*N. hyalosporus*	China, Guangxi Zhuang Autonomous Region	decaying wood	freshwater	210–290 × 3–4 μm	14–20 μm diam. 120–140 × 1.5–2.5 μm	9–20 × 3–4 μm	tightly coiled 2¹/2–3³/4 times	Lu et al. (2018)
*N. hydei*	China, Guizhou Province	decaying wood	freshwater	262–410 × 5.5–7 μm	up to 18.5 μm diam. 137.5–171.5 × 2–3 μm	7.5–19.5 × 3.5–6 μm	coiled up to 4 times	Ma et al. (2024a)
*N. kevinianus*	China, Guizhou Province	decaying wood	freshwater	3.5–6 µm wide	14–22 μm diam. 101–147 µm long	8–25 × 3–5.5 µm	tightly coiled 2¹/2–3²/3 times	Xiao et al. (2025)
*N. lignicola*	China, Hainan Province	decaying wood	freshwater	116–171 × 4–5 μm	12.5–24.5 μm diam. 98–177 × 2–4 μm	12–15 × 3–4 μm	tightly coiled up to 3¹/2 times	Ma et al. (2024b)
*N. longisetosus*	China, Taiwan Province	decaying culm of *Miscanthus floridulus* (Poaceae)	freshwater	22–30.5 × 3–3.5 μm	20–24 μm diam. 23–28–septate, 2–3.5 μm thick	1.5–2 × 1–2 μm	coiled 3–3.5 times	Hsieh et al. (2021)
*N. macrosporus*	China, Hainan Province	decaying wood	freshwater	35–86 × 4.5–6.5 μm	45–70 μm diam. 176.5–327.5 × 4.5–7.5 μm	12–23 × 3–5.5 μm	loosely coiled 1¹/4–1³/4 times	Ma et al. (2024b)
*N. maolanensis*	China, Guizhou Province	decaying wood	terrestrial	201–230 × 3–4.5 μm	13.5–19 μm diam. 105–134 × 2.5–3 μm	13.5–18.5 × 2.5–4 μm	tightly coiled 3–3³/4 times	Peng et al. (2025)
*N. melaleucae*	USA, California	leaves of *Melaleuca styphelioides × lanceolata* (Myrtaceae)	terrestrial	Conidiophores reduced to conidiogenous cells, 3–15 × 3–4 µm, at times reduced to a single denticles directly on hyphae.	13–17 µm diam. base truncate, 2 µm diam.	Conidiophores reduced to conidiogenous cells, 3–15 × 3–4 µm, with one to several flat–tipped denticles, 2 µm diam.; at times reduced to a single denticles directly on hyphae.	coiled in 3 rings	Crous et al. (2019b)
*N. pallidus*	China (Guangdong, Hebei, Liaoning, Xizang Autonomous Region, Hong Kong), the Czech Republic, Italy, Japan, Spain, the United States, the Netherlands	Decaying wood, roots of *Holcus lanatus*, *Quercus robur*, decayed and fallen branch	freshwater terrestrial	up to 580 µm long, 1.5–4 µm thick	10–15 µm diam. the filaments 1 µm in diameter		coiled 2–3¹/2 times	Linder (1929); Goos (1989); Tsui et al. (2006); Zhao et al. (2007); Sánchez Márquez et al. (2010); Lu et al. (2018); Ma et al. (2024b)
*N. pandanicola*	China, Yunnan Province	*Pandanus* sp.	terrestrial	110–220 × 3–6 μm	28–44 μm diam. 60–123 × 2–3 μm		filament coiled 2¹/2–3¹/2 times	Tibpromma et al. (2018)
*N. puerensis*	China, Yunnan Province	*Coffea arabica*	terrestrial	138.8–211.7 × 2.6–4.2 μm	15.2–22 μm diam. 106.6–211.2 × 1.9–3.7 μm	11.6–20 × 2.4–4.1 μm	tightly coiled 2.5–4.25 times	Han et al. (2026)
*N. qixingyaensis*	China, Guangxi Zhuang Autonomous Region	decaying wood	terrestrial	94.5–187 × 3.5–6 μm	16–18.5 μm diam. 98–147 × 1.5–3 μm	7–18 × 3–5.5 μm	tightly coiled 3–3¹/4 times	Ma et al. (2024b)
*N. saprobicus*	China, Guizhou Province	decaying wood	freshwater	126–160 × 4.5–6.5 μm	25–39 μm diam. 140–195 × 2.5–3.5 µm	7–18 × 3.0–5.0 µm	loosely coiled 1³/4–3 times	This study
*N. sexualis*	China, Guizhou Province	bamboo culms	terrestrial					Sun et al. (2025)
*N. submersus*	China, Yunnan Province	decaying wood	freshwater	172–285 × 3.5–4.5 μm	142.5–207.5 × 2.5–3.5 μm		tightly coiled 3–3¹/2 times	Luo et al. (2017)
*N. subtropicus*	China, Guizhou Province	decaying wood	terrestrial	up to 420 μm long, 2.5– 5.5 μm wide	14.5–16.5 μm diam. 87–132 × 1.5–3 μm	10.5–19.5 × 2–5.5 μm	tightly coiled 2–2³/4 times	Peng et al. (2025)
*N. terrestris*	China, Hainan Province	decaying wood	terrestrial	106–212 × 3–4.5 μm	15–21 μm diam. 107–143 × 2.5–3.5 μm	7.5–25 × 2–4 μm	loosely coiled 2¹/2–3 times	Gao et al. (2025)
*N. thailandicus*	Thailand, Chiang Rai Province	decaying wood	freshwater	300–400 × 2.8–4.6 µm	14–18 µm diam. 120–145 × 1.3–1.8 µm	8–11 × 2–3.5 µm	tightly coiled 3.75–4.25 times	Dong et al. (2020)
*N. tropicus*	China, Hainan Province	decaying wood	terrestrial	139–175 × 4–5.5 μm	15.5–20 μm diam. 95–140 × 2–3.5 μm	10–17 × 3–4 μm	tightly coiled up to 3 times	Gao et al. (2025)
*N. uniramulosus*	China, Guizhou Province	decaying wood	terrestrial	144–220 × 3.5–7.0 μm	13–23 μm diam. 115–166 × 2.2–3.4 µm	8–18 × 3–5 µm	tightly coiled 3¹/3–34/5 times	This study
*N. wuzhishanensis*	China, Hainan Province	decaying wood	freshwater	92–190 × 3.5–5 μm	23–26 μm diam. 118–143.5 × 2.3–3.5 μm	9.5–16.5 × 2.5–5 μm	tightly coiled 1.5–2 times	Ma et al. (2025)
*N. xiayadongensis*	China, Xizang Autonomous Region	decaying wood	terrestrial	78–126 × 3–5 μm	21–24 μm diam. 80.5–123 × 2–2.5 μm	17–19.5 × 2.5–3.5 μm	loosely coiled 1¹/5–2 times	Ma et al. (2024b)
*N. yunnanensis*	China, Yunnan Province	decaying wood	freshwater	up to 178 μm long, 6 μm wide	18–26.5 μm diam. 128.5–162 × 2.5–3.5 μm	14.5–19.5 × 3–5.5 μm	tightly coiled 3–3¹/4 times	Ma et al. (2024b)

In *Neohelicomyces*, the reported host range spans multiple plant families, reflecting a certain degree of host diversity, with documented hosts including *Holcus lanatus* (Sánchez Márquez et al. 2010), *Pandanus* sp. (Tibpromma et al. 2018), *Deschampsia cespitosa* (Poaceae) (Crous et al. 2019a), leaves of *Melaleuca styphelioides* × *lanceolata* (Myrtaceae) (Crous et al. 2019b), *Miscanthus floridulus* (Poaceae) (Hsieh et al. 2021), *Edgeworthia chrysantha* (Ma et al. 2024b), *Coffea arabica* (Han et al. 2026), *Quercus robur*, and bamboo culms (Sun et al. 2025). With the exception of *N. melaleucae*, which parasitizes the leaves of *Melaleuca styphelioides* × *lanceolata*, and *N. pallidus*, which has been isolated from the roots of *Holcus lanatus* (Sánchez Márquez et al. 2010; Crous et al. 2019b), all other *Neohelicomyces* species associated with the aforementioned hosts are considered saprobic. These saprobes are predominantly found on decaying plant material, including rotting wood, dead twigs, culms, and leaves (Luo et al. 2017; Crous et al. 2019a, 2019b; Hsieh et al. 2021; Ma et al. 2024a; Ma et al. 2025; Han et al. 2026). This suggests that decomposing plant tissue represents the primary ecological niche and survival substrate for the majority of species within the genus.

## Supplementary Material

XML Treatment for Neohelicomyces saprobicus

XML Treatment for Neohelicomyces uniramulosus

## Additional information

### Use of AI

No use of AI was reported.

### Author ORCIDs

Gui-Li Zhao https://orcid.org/0000-0001-8370-4844

Xing-Juan Xiao https://orcid.org/0009-0003-8769-4534

Shan-Shan Song https://orcid.org/0009-0009-4229-7627

Samantha C. Karunarathna https://orcid.org/0000-0001-7080-0781

Xue-Yan Chen https://orcid.org/0009-0001-7892-3417

Ying Liu https://orcid.org/0009-0003-4850-4929

Yong-Zhong Lu https://orcid.org/0000-0002-1033-5782

Ning-Guo Liu https://orcid.org/0000-0002-9169-2350

### Data availability

All of the data that support the findings of this study are available in the main text.
